# Tranexamic Acid Use in Neck of Femur Fractures: Impact on Blood Loss and Clinical Outcomes at a UK Teaching Hospital

**DOI:** 10.7759/cureus.95672

**Published:** 2025-10-29

**Authors:** Kimberly V Ponsworno, Anurag Sinha, Henry Burnand

**Affiliations:** 1 Trauma and Orthopaedics, University Hospitals Bristol and Weston NHS Foundation Trust, Bristol, GBR

**Keywords:** 30-day mortality, blood transfusion, neck of femur fractures, perioperative tranexamic acid, postoperative blood loss, post-operative outcome, thromboembolic events, tranexamic acid

## Abstract

Background

Patients with neck of femur (NOF) fractures experience high morbidity and transfusion rates. Tranexamic acid (TXA) routinely reduces perioperative blood loss in elective lower limb arthroplasties; however, its use in fragility fracture management remains variable, and there is no nationally agreed guideline for its use in hip fracture surgery. This study aims to evaluate the impact of TXA administration on transfusion rates, postoperative hemoglobin drop, in-hospital complications, and 30-day mortality in patients with NOF fractures at a UK-based teaching hospital.

Methods

A retrospective audit at a university trauma unit evaluated TXA use in patients with NOF fractures from July to December 2024. Of 184 cases identified, 26 were excluded (preoperative death, transfer, or non-NOF fractures), leaving 158 patients for analysis. Patients were grouped based on TXA administration. The primary outcome was the need for blood transfusion intraoperatively or within one week postoperatively. Secondary outcomes included postoperative hemoglobin drop, thromboembolic events, and 30-day mortality.

Results

Among 158 patients, 144 (91.1%) received TXA. Transfusion rates were significantly lower in the TXA group (22.2%) compared with non-TXA patients (50.0%) (χ² = 5.30, p = 0.021). Mean postoperative hemoglobin drop was smaller with TXA (17.1 ± 13.4 g/L vs. 22.8 ± 17.2 g/L; Z = −2.34, p = 0.019). Binary logistic regression identified TXA as an independent protective factor (OR: 0.164, 95% CI: 0.042-0.648, p = 0.011), while female sex, American Society of Anesthesiologists (ASA) grade III-IV, and extracapsular fractures were independent predictors of transfusion. There was no significant difference in thromboembolic events (3.5% vs. 7.1%, p = 0.432) or 30-day mortality (6.3% vs. 21.4%, p = 0.095), suggesting TXA did not increase these risks.

Conclusion

In this cohort, perioperative TXA use in operative NOF fracture management significantly reduced transfusion rates and hemoglobin drop without a significant effect on 30-day mortality and no effect on thromboembolic events. These findings support routine TXA use as a low-risk, cost-effective adjunct in the surgical management of patients with NOF fractures.

## Introduction

Neck of femur (NOF) fractures are increasingly common due to an aging population and are associated with high morbidity, mortality, and transfusion rates [1]. Despite efforts such as fracture liaison services and osteoporosis management to reduce fragility fractures, their incidence, particularly of the hip, continues to rise [2,3].

These fractures are associated with both short- and long-term complications. In the context of this study, short-term concerns include perioperative blood loss and thromboembolic events, while long-term complications are largely related to immobility, such as infection, pressure ulcers, and functional decline [4].

Blood loss contributes significantly to morbidity in this population. The average blood loss from NOF surgery is substantial and often underestimated due to “hidden” blood loss from both the fracture itself and the operative procedure [1]. One study found a mean postoperative hemoglobin drop of 14 ± 10.3 g/L, with over half of the patients already on anticoagulation therapy [1]. Additionally, most patients presenting with NOF fractures are American Society of Anesthesiologists (ASA) grade III or IV and often have multiple comorbidities [5]. Many are anemic at baseline, which can impair mobility and is an independent risk factor for delayed postoperative recovery and poorer outcomes [6]. This makes the prevention of further blood loss a key clinical goal.

Tranexamic acid (TXA) is a synthetic antifibrinolytic agent that inhibits the conversion of plasminogen to plasmin, thereby stabilizing fibrin clots and reducing bleeding. It is routinely used in elective total joint arthroplasty to reduce perioperative blood loss without increasing the risk of venous thromboembolism (VTE) [7]. However, its use in the fragility fracture population remains inconsistent, and there is currently no national guideline on TXA use in hip fracture surgery. This variability in clinical practice highlights a knowledge-to-practice gap that may limit the consistent application of TXA’s benefits in this vulnerable population.

Emerging evidence, including multiple studies and meta-analyses, has demonstrated that TXA is effective in reducing blood loss and transfusion requirements in hip fracture patients, without significantly increasing thromboembolic complications [7-10]. According to local guidelines, 1 g of TXA is administered via slow intravenous (IV) injection at induction for all NOF fracture surgeries, with dose adjustments made based on renal function and body weight (standard dose 15 mg/kg, maximum 1 g for patients weighing less than 66 kg) [11]. This study evaluates the impact of TXA administration on transfusion rates, hemoglobin drop, in-hospital thromboembolic complications, and 30-day mortality among NOF fracture patients at a UK teaching hospital, addressing the lack of national guidelines and variability in TXA use.

This article has been submitted as an abstract to the 21st Annual Academic Surgical Congress (ASC) and 27th EFORT Congress in 2026, but has not yet been presented.

## Materials and methods

Study design

This retrospective observational study was conducted at a university trauma unit in the United Kingdom and included all patients admitted with radiologically confirmed NOF fractures between July 1 and December 31, 2024.

Patient selection

**Figure 1:**
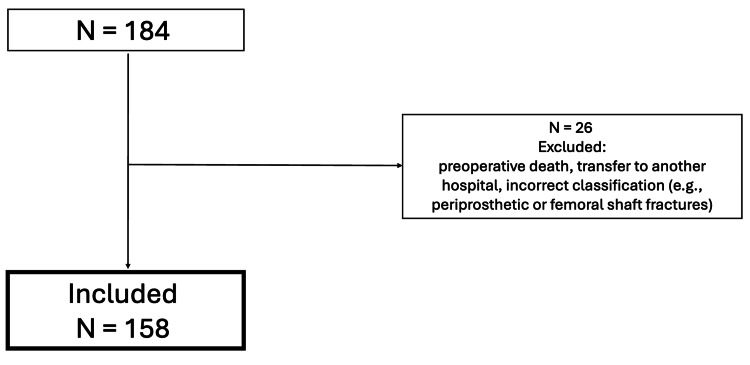
Inclusion and exclusion criteria.

A total of 184 patients were identified during the study period. Inclusion criteria were patients aged ≥18 years with radiologically confirmed NOF fractures who underwent operative management. Exclusion criteria included periprosthetic or femoral shaft fractures (n = 15), preoperative death (n = 3), transfer to another hospital prior to surgery (n = 4), and incomplete records preventing confirmation of TXA administration or outcome measures (n = 4). Twenty-six patients were excluded, leaving 158 for the final analysis (Figure 1).

Patients were grouped according to whether they received intraoperative TXA. For statistical analysis, the standard and variable dose groups were combined into a single TXA group (n = 144; 91.1%) and compared with the non-TXA group (n = 14; 8.9%), given the small number and similar dosing strategies of the variable dose subgroup.

Preoperative risk was assessed using the ASA physical status classification system, which ranges from ASA I (a healthy patient) to ASA VI (a brain-dead patient undergoing organ donation). Patients were categorized as ASA I-II (low to moderate risk) or ASA III-IV (high risk) [5].

Outcomes

The primary outcome was the requirement for blood transfusion, either intraoperatively or within one week postoperatively. Secondary outcomes included the change in hemoglobin from pre- to postoperative measurements, the occurrence of thromboembolic events (including deep vein thrombosis, pulmonary embolism, myocardial infarction, cerebrovascular accident, and peripheral limb ischemia), and 30-day mortality. Preoperative hemoglobin levels were obtained within one day prior to surgery, while postoperative hemoglobin was measured using HemoCue in the recovery area or from the first postoperative full blood count on day one. Blood transfusion was indicated for postoperative acute anemia in hemodynamically stable patients with a hemoglobin threshold of 70 g/L, in accordance with local transfusion guidelines.

Data collection

Clinical data were obtained from electronic hospital records and entered into Microsoft Excel 2024 (Microsoft Corporation, Redmond, WA). TXA administration and dosing were confirmed from scanned anesthetic charts accessed via the Evolve platform, while laboratory values and transfusion records were obtained from the Integrated Clinical Environment system. Inpatient notes, discharge summaries, and imaging reports were reviewed for thromboembolic events and 30-day mortality. Only complete cases were analyzed.

Statistical analysis

Descriptive statistics summarized patient demographics, clinical characteristics, and outcomes. The Shapiro-Wilk test assessed the normality of continuous variables. Categorical variables, including transfusion, thromboembolic events, and mortality, were compared using chi-square or Fisher’s exact tests, while non-normally distributed continuous variables, such as hemoglobin drop, were analyzed with the Mann-Whitney U test. Univariate analyses were conducted using Social Science Statistics [12].

Given disparities in gender and ASA distribution, a multivariate binary logistic regression model was performed using SPSS version 31.0 (IBM Corp., Armonk, NY) to identify independent predictors of blood transfusion, the study’s primary outcome. Covariates included age group (<75 or ≥75 years), sex, ASA grade (I-II or III-IV), and fracture type (intracapsular or extracapsular). The dependent variable was blood transfusion requirement (yes/no), and the main independent variable was TXA administration. Procedure type was analyzed descriptively but excluded from the regression model due to its strong correlation with fracture type and the risk of model overfitting.

Multivariate analysis was limited to the primary outcome to maintain statistical power and avoid overadjustment. Secondary outcomes (hemoglobin drop, thromboembolic events, and 30-day mortality) were therefore analyzed descriptively using univariate tests. A p-value <0.05 was considered statistically significant.

Ethical considerations

The study was registered locally as a clinical audit and conducted in accordance with institutional research governance guidelines. As anonymized data were collected retrospectively without direct patient contact, formal ethical approval was not required.

## Results

Patient characteristics

A total of 158 patients were included, of whom 144 (91.1%) received TXA and 14 (8.9%) did not. The mean age was 79 ± 13.0 years in the TXA group and 68 ± 18.3 years in the non-TXA group. Most patients were aged ≥75 years (68.8% vs. 42.9%), and females comprised 61.1% and 57.1% of each group, respectively. A majority were high-risk ASA grade III-IV (72.2% vs. 64.3%), and fracture distribution was similar between groups (extracapsular 45.8% vs. 42.9%). Patient demographics, ASA classification, and fracture characteristics are summarized in Table 1.

**Table 1:** Patient demographics and clinical characteristics by TXA use. TXA: tranexamic acid; SD: standard deviation.

Characteristics	TXA (n = 144)	Non-TXA (n = 14)
Mean age (years) ± SD	79 ± 13.0	68 ± 18.3
<75 years	45 (31.2%)	8 (57.1%)
≥75 years	99 (68.8%)	6 (42.9%)
Sex		
Female	88 (61.1%)	8 (57.1%)
Male	56 (38.9%)	6 (42.9%)
American Society of Anesthesiologists (ASA) grade		
I-II	40 (27.8%)	5 (35.7%)
III-IV	104 (72.2%)	9 (64.3%)
Fracture type		
Intracapsular	78 (54.2%)	8 (57.1%)
Extracapsular	66 (45.8%)	6 (42.9%)

Among the TXA recipients, 131 patients (81.9%) received 1 g IV at induction per local protocol. The remaining 13 patients (8.2%) received variable dosing regimens, either a reduced dose based on renal function or body weight, or an additional dose at closure in response to intraoperative blood loss at the surgeon's discretion. Variable dosing strategies included the following: (1) 1 g IV at induction + 1 g IV at closure; (2) 750 mg IV at induction; (3) 500 mg IV at induction; (4) 500 mg IV at induction + 500 mg IV at closure; and (5) 1 g IV at induction + 500 mg IV at closure.

Patient outcomes (univariate analysis)

Transfusion Rate

Within one week postoperatively, 22.2% of patients in the TXA group (32/144) required blood transfusion compared to 50.0% in the non-TXA group (7/14). This difference was statistically significant (χ² = 5.296, p = 0.021), indicating an association between TXA use and reduced transfusion requirement.

Hemoglobin Drop

The mean postoperative hemoglobin drop was significantly lower in patients who received TXA (17.1 g/L) compared to those who did not (22.8 g/L), as shown by a two-tailed Mann-Whitney U test (Z = -2.34, p = 0.019).

Thromboembolic Events

Postoperative thromboembolic events occurred in five patients in the TXA group and one patient in the non-TXA group. Fisher’s exact test indicated no statistically significant difference (p = 0.432), suggesting TXA administration was not associated with increased thromboembolic risk.

Thirty-Day Mortality

The 30-day mortality rate did not differ significantly between groups (χ² = 2.78, p = 0.095). This result remained non-significant after applying Yates’ correction for continuity (χ² = 1.60, p = 0.206).

Primary and secondary outcomes are summarized in Table 2.

**Table 2:** Univariate analysis of patient outcomes by TXA administration. ᵃ Chi-square test; ᵇ Mann-Whitney U test; ᶜ Fisher’s exact test; ^d ^Yates’ correction. TXA: tranexamic acid.

Primary outcome	TXA (n = 144)	Non-TXA (n = 14)	p-value
Transfusion rate	32 (22.2%)	7 (50.0%)	0.021^a^
Secondary outcome			
Mean hemoglobin drop (g/L) ± SD	17.1 ± 13.4	22.8 ± 17.2	0.019^b^
Thromboembolic events	5 (3.5%)	1 (7.1%)	0.432^c^
30-day mortality	9 (6.3%)	3 (21.4%)	0.095^a^
			0.206^d^

Procedure-specific outcomes

TXA reduced transfusion rates and hemoglobin drop across all surgical subgroups (Table 3). Among arthroplasty cases, transfusion was required in 19.0% of the TXA group versus 42.8% without TXA. In fixation procedures, transfusion rates were 47.6% for intramedullary nailing, 7.1% for dynamic hip screw fixation, and 0% for cannulated screws in the TXA group. Mean hemoglobin drops followed similar trends, with the largest reductions observed in intramedullary nail fixation (19.0 ± 18.3 g/L for TXA versus 31.5 ± 7.1 g/L for non-TXA) and dynamic hip screw fixation (15.3 ± 15.4 g/L for TXA versus 29.5 ± 14.9 g/L for non-TXA). Hemiarthroplasty patients had moderate drops, while total hip arthroplasty and cannulated screw subgroups showed smaller reductions.

**Table 3:** Transfusion rates and mean hemoglobin drop by procedure type and TXA administration. * For non-TXA subgroups with a single case (total hip arthroplasty and cannulated screws), the standard deviation could not be calculated. TXA: tranexamic acid.

Procedure	TXA (n = 144)			Non-TXA (n = 14)		
	n (%)	Transfusion rate (n = 32)	Mean hemoglobin drop (g/L)	n (%)	Transfusion rate (n = 7)	Mean hemoglobin drop (g/L)
Arthroplasty						
Hemiarthroplasty	56 (38.9%)	8 (19.0%)	17.0 ± 8.7	4 (28.6%)	3 (42.8%)	19.5 ± 16.3
Total hip arthroplasty	8 (5.6%)	1 (2.4 %)	17.6 ± 9.3	1 (7.1%)	0 (0%)	18.0 (SD not applicable)*
Fixation						
Cannulated screws	13 (9.0%)	0 (0%)	12.8 ± 6.4	1 (7.1%)	0 (0%)	22.0 (SD not applicable)*
Dynamic hip screw	21 (14.6%)	3 (7.14%)	15.3 ± 15.4	4 (28.6%)	2 (28.6%)	29.5 ± 14.9
Intramedullary nail	46 (31.9%)	20 (47.6%)	19.0 ± 18.3	4 (28.6%)	2 (28.6%)	31.5 ± 7.1

These findings indicate that TXA reduced blood loss across procedure types; however, fixation of extracapsular fractures, particularly intramedullary nailing, remained associated with higher transfusion rates and hemoglobin drops, reflecting the greater intraoperative blood loss inherent to these procedures.

Multivariate analysis

The binary logistic regression model was statistically significant compared with the null model, χ²(5) = 32.57, p < 0.001, explaining 28.4% of the variance in transfusion requirement (Nagelkerke R² = 0.284) and correctly classifying 76.6% of cases.

The regression model for the primary outcome identified TXA administration, sex, ASA grade, and fracture type as independent predictors of blood transfusion requirement (Table 4).

**Table 4:** Binary logistic regression analysis for predictors of blood transfusion requirement. ^a ^Adjusted odds ratio. ^b ^95% confidence interval for the odds ratio. TXA: tranexamic acid; ASA: American Society of Anesthesiologists.

Independent variables	p-value	Adjusted OR^a^	95% CI for EXP(B)^b^	
			Lower	Upper
TXA	0.01	0.164	0.042	0.648
Age range (<75, ≥75)	0.712	1.208	0.442	3.301
Sex (male, female)	0.004	4.049	1.548	10.596
ASA (I-II, III-IV)	0.026	3.738	1.168	11.966
Fracture type (intracapsular, extracapsular)	0.002	3.805	1.63	8.885

Among the included covariates, TXA administration (p = 0.010), sex (p = 0.004), ASA grade (p = 0.026), and fracture type (p = 0.002) were statistically significant predictors of transfusion, while age group (<75 vs. ≥75 years) was not (p = 0.712). Patients who did not receive TXA were approximately six times more likely to require transfusion (OR = 0.164, 95% CI: 0.042-0.648). Female patients were four times more likely than males to receive a transfusion (OR = 4.049, 95% CI: 1.548-10.596). Patients with ASA grade III-IV were 3.7 times more likely to require transfusion than those with ASA I-II (OR = 3.738, 95% CI: 1.168-11.966). Extracapsular fractures were associated with a 3.8-fold increased risk of transfusion compared with intracapsular fractures (OR = 3.805, 95% CI: 1.630-8.885).

These findings indicate that, while patient- and fracture-related characteristics influence transfusion risk, TXA administration remains a significant and modifiable protective factor against perioperative blood transfusion.

## Discussion

Blood loss associated with NOF fractures is influenced by both non-modifiable factors (fracture pattern, bleeding disorders, pre-existing anticoagulant or antiplatelet use) and modifiable factors (surgeon grade, preoperative anemia, timing of withholding anticoagulation, intraoperative complications, and perioperative use of TXA). This study focuses on the latter, specifically evaluating the effectiveness of perioperative TXA in minimizing blood loss and transfusion.

TXA administration at induction was associated with a significant reduction in transfusion requirement and remained an independent protective factor on multivariate analysis. This aligns with Lei et al., who reported reduced transfusion rates in intertrochanteric fractures treated with proximal femoral nail anti-rotation fixation [8], and with the multicenter PATHS audit, which demonstrated lower transfusion rates with perioperative TXA use across UK centers [10]. Other independent predictors included female sex, higher ASA grade (III-IV), and extracapsular fracture type. Female patients have previously been shown to have higher transfusion risk, attributed to lower baseline hemoglobin and circulating volume [13]. Higher ASA grade reflects frailty and comorbidity burden, correlating with greater transfusion likelihood [14], while extracapsular fractures typically result in greater soft tissue bleeding due to their anatomy [15]. Age was not independently associated with transfusion risk, consistent with Desai et al., suggesting that other patient and fracture factors are more predictive [16].

TXA recipients experienced a smaller postoperative hemoglobin drop, with benefits persisting across surgical subgroups, including high blood loss procedures such as intramedullary nailing [17]. These findings mirror those of Xie and Himeno, who reported reduced transfusion rates and hemoglobin decline with TXA use in geriatric hip fracture patients [18].

Importantly, no statistically significant increase in thromboembolic complications (deep vein thrombosis, pulmonary embolism, acute coronary syndrome, or stroke) was observed within 30 days among TXA recipients. These outcomes align with meta-analyses by Zhang et al., confirming no elevated VTE risk with TXA [9], and with Viberg et al., who found no association between TXA use and short- or mid-term mortality [19].

Despite demonstrated benefits, perioperative TXA use remains inconsistent. A local survey of 18 clinicians (11 orthopedic surgeons and seven anesthetists) revealed that 89% were unaware of the unit’s TXA guideline for NOF fractures, and 22% reported they would not routinely administer TXA. This knowledge-to-practice gap may limit the clinical benefits observed and highlights the need for improved dissemination of protocols and education among perioperative teams.

Current literature describes multiple administration strategies with differing efficacies and timing [9,10]. Lasocki et al. outlined various routes and dosing regimens for TXA in hip fracture surgery, including intravenous, topical, and combined approaches [20]. In this study, most patients received 1 g IV TXA at induction, although a few cases varied in timing or dosing according to weight and renal function, consistent with local policy. The National Institute for Health and Care Excellence (NICE) recommends IV TXA in major trauma within three hours of injury and in surgeries with expected blood loss >500 mL, in line with CRASH-2 findings [21,22]. The British National Formulary advises unlicensed use of a 1 g IV loading dose within eight hours of injury, followed by a 1 g infusion over eight hours, for prevention and treatment of significant hemorrhage [23]. However, no national guideline exists specifically for hip fractures, and dosing practices vary across UK centers [10]. Further evidence supports weight- and renal-adjusted regimens [11].

A potential avenue for future research is that early TXA administration in the emergency department may offer additional benefit by reducing hidden preoperative blood loss, particularly in frail elderly patients with limited physiological reserve, as described by Stacey et al. [1].

Limitations

This study is limited by its retrospective design, single-center setting, and small non-TXA group, which may limit statistical power. The predominance of female patients and a higher proportion of ASA III-IV cases may also introduce selection bias, though regression analysis was used to adjust for these factors. Potential confounders such as surgeon experience, anesthetic technique, intraoperative blood pressure control, and fracture morphology were not standardized. Reliance on existing records may have introduced information bias due to incomplete documentation. Future prospective multicenter studies with standardized TXA dosing and timing are warranted to validate these findings.

## Conclusions

Blood loss in NOF fracture patients is multifactorial. In this study, IV TXA given at induction significantly reduced postoperative transfusion rates and mean hemoglobin drop without increasing the risk of VTE or 30-day mortality. These findings support TXA as a safe and effective adjunct in the surgical management of NOF fractures. Adherence to local protocols should be reinforced, and further large-scale, controlled studies are needed to establish a national guideline on TXA use in this patient population.

## References

[1] Stacey J, Bush C, DiPasquale T (2021). The hidden blood loss in proximal femur fractures is sizeable and significant. J Clin Orthop Trauma.

[2] (2025). A broken hip - three steps to recovery. The 2024 National Hip Fracture Database report on 2023. https://www.nhfd.co.uk/20/hipfractureR.nsf/docs/2024Report.

[3] Naranjo A, Fernández-Conde S, Ojeda S (2017). Preventing future fractures: effectiveness of an orthogeriatric fracture liaison service compared to an outpatient fracture liaison service and the standard management in patients with hip fracture. Arch Osteoporos.

[4] Dyer SM, Crotty M, Fairhall N, Magaziner J, Beaupre LA, Cameron ID, Sherrington C (2016). A critical review of the long-term disability outcomes following hip fracture. BMC Geriatr.

[5] Hendrix JM, Garmon EH (2025). American Society of Anesthesiologists physical status classification system. StatPearls.

[6] Fenwick A, Pfann M, Mayr J (2023). Anticoagulants and fracture morphology have a significant influence on total blood loss after proximal femur fractures. Eur J Trauma Emerg Surg.

[7] Anna K, Röttinger T, Lisitano L, Koenemann N, Förch S, Mayr E, Fenwick A (2025). Tranexamic acid: single topical application for femoral neck fractures treated with arthroplasty results in lowest blood loss. Eur J Trauma Emerg Surg.

[8] Lei J, Zhang B, Cong Y (2017). Tranexamic acid reduces hidden blood loss in the treatment of intertrochanteric fractures with PFNA: a single-center randomized controlled trial. J Orthop Surg Res.

[9] Zhang J, Fan X, Zheng Y, Wu J, Yuan X (2023). Intravenous application of tranexamic acid in intramedullary nailing for the treatment of geriatric intertrochanteric fractures: a systematic review and meta-analysis. BMC Musculoskelet Disord.

[10] Berg AJ, Naylor T, Johnson DS (2023). Perioperative administration of tranexamic acid in hip fracture surgery (the PATHS study): national audit of current practice. Ann R Coll Surg Engl.

[11] (2025). Cyklokapron 100 mg/mL solution for injection/infusion. https://www.medicines.org.uk/emc/product/1077/smpc.

[12] (2025). Social Science Statistics. https://www.socscistatistics.com/.

[13] Smith GH, Tsang J, Molyneux SG, White TO (2011). The hidden blood loss after hip fracture. Injury.

[14] Goodman M, Pillai A (2024). A comparative analysis of age, BMI, age/BMI ratio, Nottingham Hip Fracture Score, and American Society of Anesthesiologists (ASA) grade as predictors of 30-day mortality after neck of femur fractures: a retrospective cohort study. Cureus.

[15] Testa G, Montemagno M, Vescio A (2023). Blood-transfusion risk factors after intramedullary nailing for extracapsular femoral neck fracture in elderly patients. J Funct Morphol Kinesiol.

[16] Desai SJ, Wood KS, Marsh J, Bryant D, Abdo H, Lawendy AR, Sanders DW (2014). Factors affecting transfusion requirement after hip fracture: can we reduce the need for blood?. Can J Surg.

[17] Alessio-Mazzola M, Traverso G, Coccarello F, Sanguineti F, Formica M (2022). Dynamic hip screw versus intramedullary nailing for the treatment of A1 intertrochanteric fractures: a retrospective, comparative study and cost analysis. Jt Dis Relat Surg.

[18] Xie J, Himeno S (2024). Tranexamic acid efficacy in geriatric hip fractures: impact of nutritional status on blood loss, transfusion rates, and safety. BMC Musculoskelet Disord.

[19] Viberg B, Gundtoft PH, Schønnemann JO (2021). Is tranexamic acid use in patients with a hip fracture safe?. Bone Joint J.

[20] Lasocki S, Campfort M, Léger M, Rineau E (2025). Tranexamic acid in hip fracture repair surgery: safe and effective?. Blood Transfus.

[21] National Clinical Guideline Centre (UK) (2016). Major Trauma: Assessment and Initial Management.

[22] CRASH-2 Trial Collaborators (2010). Effects of tranexamic acid on death, vascular occlusive events, and blood transfusion in trauma patients with significant haemorrhage (CRASH-2): a randomised, placebo-controlled trial. Lancet.

[23] (2025). British National Formulary. Tranexamic acid. https://bnf.nice.org.uk/drugs/tranexamic-acid/.

